# Combining airborne laser scanning and Landsat data for statistical modeling of soil carbon and tree biomass in Tanzanian Miombo woodlands

**DOI:** 10.1186/s13021-017-0076-y

**Published:** 2017-04-17

**Authors:** Mikael Egberth, Gert Nyberg, Erik Næsset, Terje Gobakken, Ernest Mauya, Rogers Malimbwi, Josiah Katani, Nurudin Chamuya, George Bulenga, Håkan Olsson

**Affiliations:** 1 Department of Forest Resource Management, Swedish University of Agricultural Sciences, Umeå, Sweden; 2 Department of Forest Ecology and Management, Swedish University of Agricultural Sciences, Umeå, Sweden; 3 Department of Ecology and Natural Resource Management, Norwegian University of Life Sciences, Ås, Norway; 4 Department of Forest Mensuration and Management, Sokoine University of Agriculture, Morogoro, United Republic of Tanzania; 5 Tanzania Forest Services Agency, Ministry of Natural Resources and Tourism, Morogoro, United Republic of Tanzania; 6 Department of Business Administration, Technology and Social Sciences, Luleå University of Technology, Luleå, Sweden

**Keywords:** Soil carbon, Biomass, Landsat 8 OLI, Airborne laser, Miombo woodlands

## Abstract

**Background:**

Soil carbon and biomass depletion can be used to identify and quantify degraded soils, and by using remote sensing, there is potential to map soil conditions over large areas. Landsat 8 Operational Land Imager satellite data and airborne laser scanning data were evaluated separately and in combination for modeling soil organic carbon, above ground tree biomass and below ground tree biomass. The test site is situated in the Liwale district in southeastern Tanzania and is dominated by Miombo woodlands. Tree data from 15 m radius field-surveyed plots and samples of soil carbon down to a depth of 30 cm were used as reference data for tree biomass and soil carbon estimations.

**Results:**

Cross-validated plot level error (RMSE) for predicting soil organic carbon was 28% using only Landsat 8, 26% using laser only, and 23% for the combination of the two. The plot level error for above ground tree biomass was 66% when using only Landsat 8, 50% for laser and 49% for the combination of Landsat 8 and laser data. Results for below ground tree biomass were similar to above ground biomass. Additionally it was found that an early dry season satellite image was preferable for modelling biomass while images from later in the dry season were better for modelling soil carbon.

**Conclusion:**

The results show that laser data is superior to Landsat 8 when predicting both soil carbon and biomass above and below ground in landscapes dominated by Miombo woodlands. Furthermore, the combination of laser data and Landsat data were marginally better than using laser data only.

**Electronic supplementary material:**

The online version of this article (doi:10.1186/s13021-017-0076-y) contains supplementary material, which is available to authorized users.

## Background

The Miombo woodlands of Tanzania are under pressure for several reasons, among them a general population increase which brings a need for subsistence agriculture as well as small scale charcoal production [1, 2]. The loss of natural ecosystems is a common pattern which occurs when subsistence agriculture increases in the transition towards an urbanized society with more intensive agriculture [3]. In the case of Tanzania, the National Forest Resources Monitoring and Assessment of Tanzania (NAFORMA; [4]) estimates that the annual consumption of forest exceeds the available resources by 19.5 million m^3^ and Hansen et al. [5] estimated a net loss of 17,000 km^2^ of forests and woodlands above 5 m height in Tanzania between the years 2000 and 2012. This deficit is currently met by overharvesting inaccessible forests and illegal harvesting in protected areas, thus diminishing the overall forest and woodland area.

Miombo woodlands are a mosaic of areas with different tree densities, often with a varying degree of degradation. The woodlands are also often mixed with agricultural fields that are covered by crops or have open soil, depending on season. There is a large number of criteria used for defining Miombo degradation [6] of which the United Nations Framework Convention on Climate Change (UNFCCC) definition is related to loss of carbon stock during a certain time period [7].

Soil organic carbon (SOC) is an important part of the soil ecosystem; the disturbance of natural forests in tropical areas, as well as the conversion of forests and woodlands to agricultural land is known to generally reduce SOC [8–12]. Traditionally soil maps have been created where soil types have been classified into taxonomic units. Land degradation is however a continuous process and it is of interest to investigate to what degree remote sensing in combination with field plot data can be an aid for following this process over time [13].

The first attempts to use remote sensing for estimation of SOC were based on the fact that soils with a higher organic matter content, i.e., higher proportion SOC, generally appear darker. This led to studies relating data from electro-optical sensing with organic matter [14–16]. Recent research covering large areas in East Africa confirms that optical satellite imagery could be used to predict SOC as well as other soil properties. Vågen et al. [10] obtained a R^2^ of 0.79 when modeling SOC in Ethiopia and Vågen and Winowiecki [17] obtained a R^2^ of 0.65 when their study material was extended to also include test sites in Kenya and Tanzania. In both studies, SOC were modeled from Landsat ETM+ data for plots of 1000 m^2^, whereas soil data were averaged from one sample from each of four subplots. Winowiecki et al. [11] subsequently obtained an R^2^ of 0.85 when modeling SOC on 166 of these plots near Lushoto, Tanzania, and using Rapid Eye optical satellite data instead of Landsat data.

There is also a need for the development of accurate methods for estimation of above ground tree biomass (AGB) and below ground tree biomass (BGB) for carbon accounting, including the measuring, reporting and verification (MRV) needed within countries’ efforts to reduce emissions from deforestation and forest degradation (REDD+) ([18–20]; http://www.un-redd.org), as well as for national and regional planning of forest resources. In the case of Tanzania, the sample based NAFORMA inventory is a key source for national level data about forests and woodlands [4, 21], but remote sensing methods used in combination with the field plots will allow estimates both for smaller areas or estimates with lower error [22].

Optical satellite data have been used for estimation of AGB since the launch of the first Landsat satellite 1972 [23]. The use of regression is one of the standard methods for modelling biomass using remote sensing data as independent variables and data from ground reference plots as dependent variables [24]. Landsat multispectral satellite data are a natural first hand choice among the remote sensing data sources, since the data are freely available, have a suitable pixel size of 30 m and wavelength bands suitable for forest monitoring, are regularly provided and offer a data continuity since the 1980s. In particular, the new Operational Land Imager (OLI) sensor onboard Landsat 8 also offers improved performance, such as better signal to noise ratios [25]. Additionally, the new European Sentinel 2 satellite system provides free optical satellite images but with more wavelength bands than Landsat [26].

As an example of early Landsat studies in dry tropical forests, Roy and Ravan [27] used Landsat TM for regression modelling of AGB in dry forest areas in India and obtained an R^2^(adj) value of 0.70 on a sample plot level. Gizachew et al. [28] modeled total tree biomass (defined as AGB + BGB) from Landsat 8 OLI data in a recent study in the Liwale district in Tanzania. The field data consisted of 500 plots from the NAFORMA inventory, distributed within an area of 15,700 km^2^. They obtained a RMSE of 49% (63% after cross validation) for plot level modeling of total tree biomass using only the Normalized Difference Vegetation Index (NDVI) from one Landsat 8 OLI image as the independent variable.

Airborne laser scanning (ALS) produces a point cloud with three dimensional coordinates for laser returns from the ground and vegetation. ALS data therefore often provide more information about tree canopies than “two dimensional” spectral data from optical satellite data and will generally provide the best data for modeling above ground tree biomass and other tree-size related variables. However, the discrete return ALS systems that are commonly used are not very reliable for estimation of vegetation near the ground. Mauya et al. [29] modeled AGB with ALS using Linear Mixed Modeling (LMM) and similar plot sizes to Gizachew et al. [28]. The obtained RMSE after cross validation was 28.4% for forests, 47.7% for woodlands, and 80.2% for other land cover types. Næsset et al. [22] investigated the use of different remote sensing data sources for a sampling study based on a subset of the plots previously used in the Liwale study area. In addition to the results related to precision for sampling based estimates, their results showed that ALS data provided the best plot level models for AGB (R^2^ = 0.64) followed by high resolution satellite images from RapidEye (R^2^ = 0.53). Use of interferometric radar data (InSAR), as well as a global Landsat product and PALSAR L-band satellite data performed less well with an R^2^ of 0.25, 0.11 and 0.05, respectively.

Important features of the three dimensional canopy structure can be derived from ALS data, while Landsat, or other similar sensors, measure reflected light in several wavelengths. Since these data sources provide complementary information, the best results can be expected to be obtained when ALS and Landsat data are used in combination. Ediriweera et al. [30] estimated AGB by combining Landsat 5 TM data and ALS data for two study areas in Australia: one subtropical rainforest area, and one Eucalyptus forest. They found that the ALS data performed better than the Landsat data for both sites. The combination of Landsat TM data and ALS data improved R^2^ for the Eucalyptus forest by 3%, but did not improve the model for the tropical rainforest.

The purpose of the current study was to compare the usefulness of Landsat 8 OLI data and ALS data, separately and in combination, for modeling of SOC, AGB and BGB in the Miombo woodlands of Tanzania.

## Methods

### Study area

**Fig. 1:**
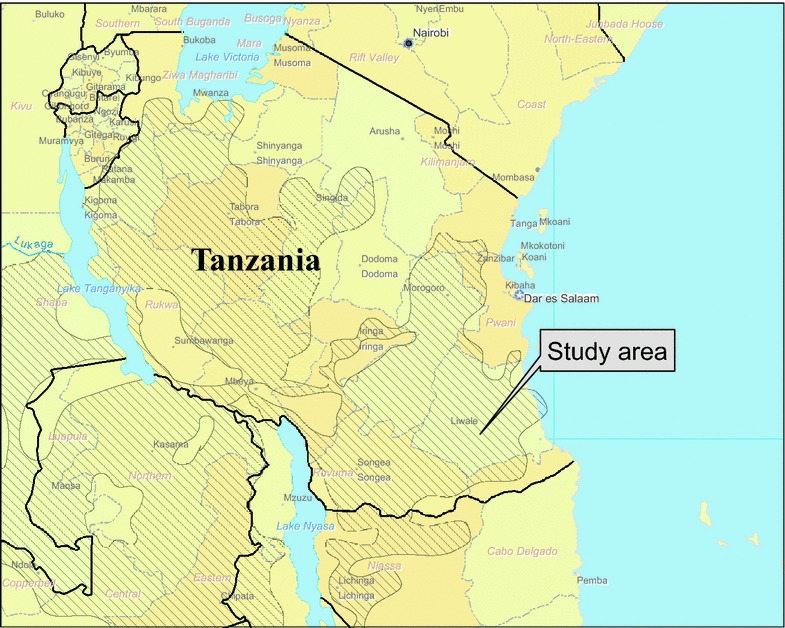
Location of the study area, the striped pattern roughly indicates Miombo woodland distribution in the area

**Fig. 2:**
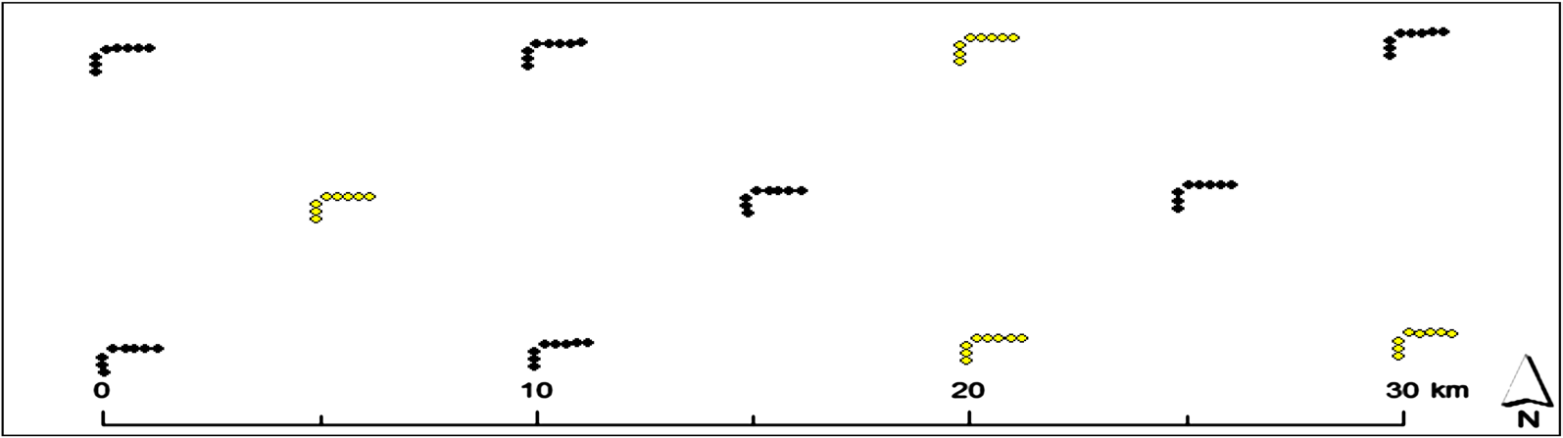
Location within the study area of the 11 clusters containing eight plots each that were used for collection of field data. The locations of four of these clusters, marked as *yellow*, are identical to the clusters used in the NAFORMA program

The study area is located in Liwale District, one of six districts of the Lindi region of southeastern Tanzania. The area is part of the Eastern Miombo Woodland ecoregion, which covers a relatively unbroken area in the interior regions of southeastern Tanzania and the northern half of Mozambique, as well as parts of southeastern Malawi. The study area is a rectangular block of 11.25 km × 32.50 km (total area 36,562 ha), and is the same area as used by Naesset et al. [22] (Figs. 1, 2).

**Fig. 3:**
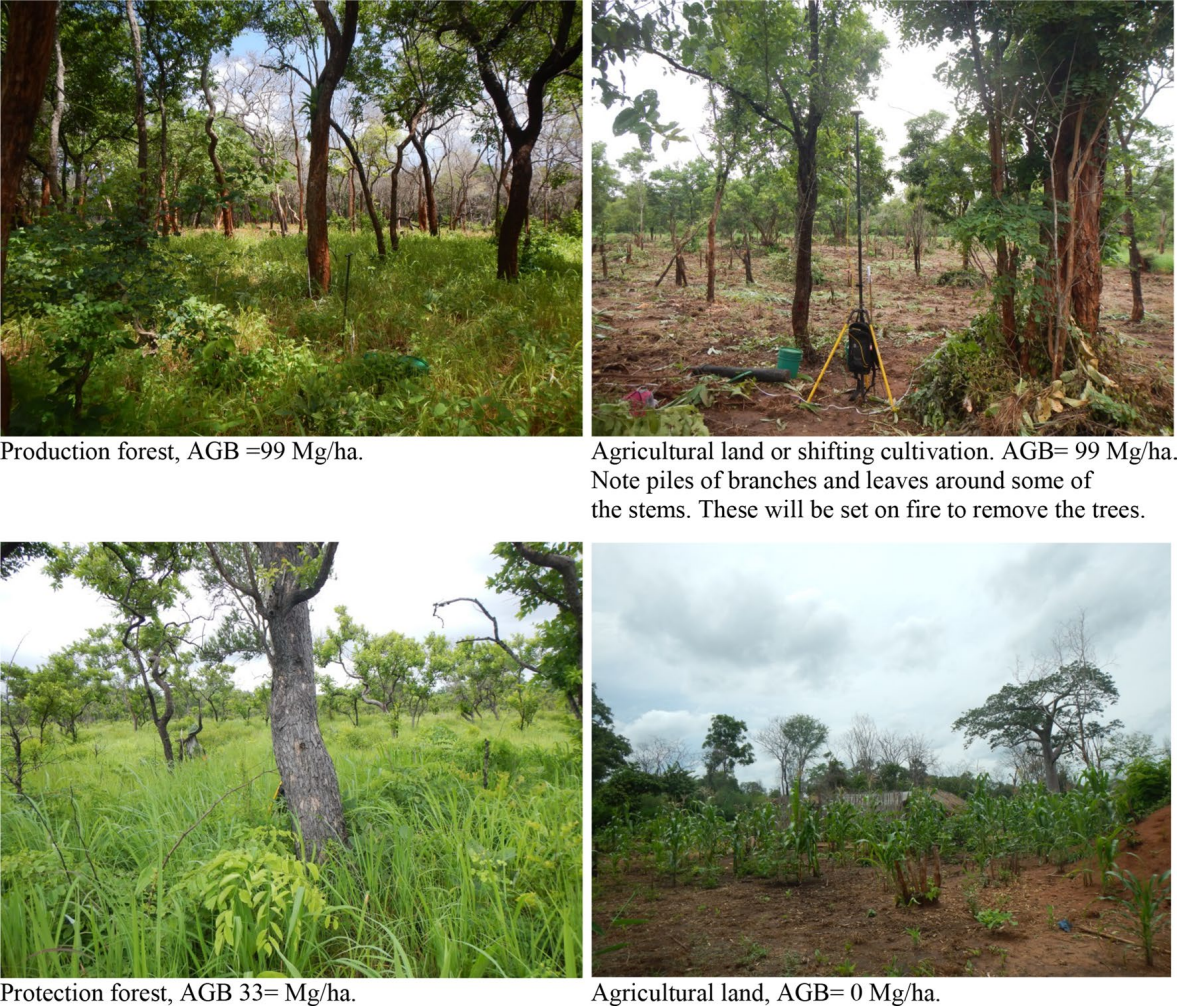
Photos from different types of land taken on measured field plots within the study area, production forest, agricultural land and shifting cultivation are land use classes defined in NAFORMA [31]

The study area consists of Miombo woodlands, mixed with shifting cultivation and permanent fields of cashew trees together with food crops (Fig. 3). In the upper left corner of the study area a forest protection area occupies approximately 4000 ha, i.e., 11% of the total study area.

The climate in the Liwale area is characterized by two rain periods a year and a main dry season. The shorter period of rain is from late November to January and the longer period is from March to May. The main dry season is between July and October. The annual precipitation is in the range 600–1000 mm. The soils in the Eastern Miombo Woodlands are relatively nutrient poor which limits the agricultural potential. There is also a widespread presence of tsetse fly (*Glossina* spp.) and vectors of *trypanosomiasis,* which affect the possibilities for settlement of both humans and livestock. However, population growth has increased demand for arable land, thus soils that in earlier years were not profitable enough are now to an increasing degree being utilized.

**Fig. 4:**
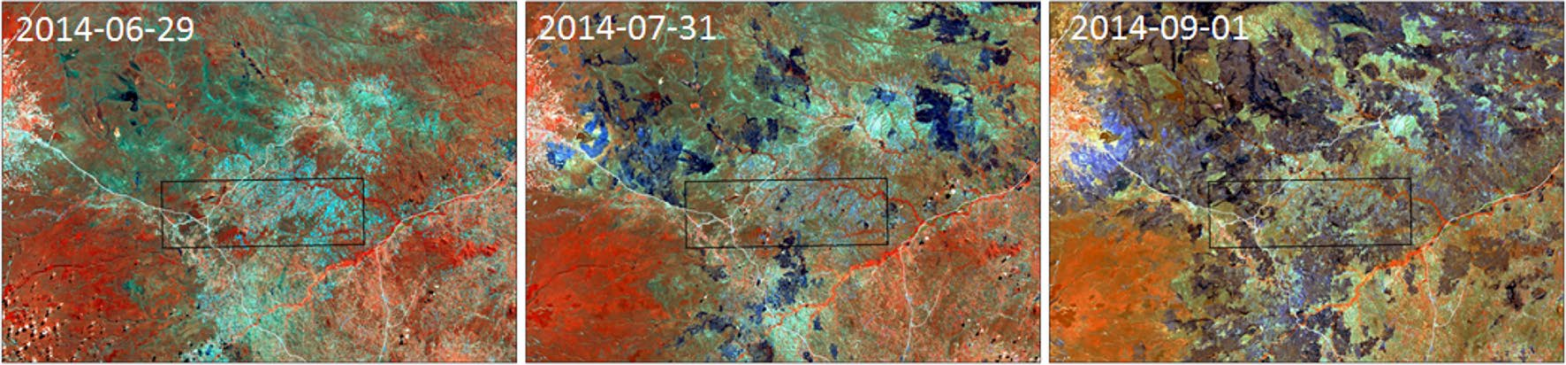
Around June, bush fires start to appear in the study area. Here illustrated in three Landsat 8 OLI images where black burnt areas clearly spread during July to September

The Miombo woodlands of Liwale are characterized by high tree species diversity including highly valuable timber species such as *Brachystegia* spp., *Julbernardia* spp. and *Pterocarpus angolensis*. According to the field sample survey conducted within the project, the study area consists of 61% forest or woodland, 14% grassland and 25% cultivated land. The definitions used are according to NAFORMA [31]. In the wooded areas human disturbances occur in the form of harvesting for timber, charcoal burning, honey collection and game hunting. Fire is also an important factor in the Miombo woodlands, underlined by the seasonality in precipitation which leaves the vegetation dry for several months. In the study area, the lack of cattle also leaves large amounts of grasses that dry and therefore are easily set alight (Fig. 4).

### Field data measurements

The field plots used as reference data were located in eleven clusters that were systematically sampled (Fig. 1). The locations of four of these clusters are identical to the clusters used in the NAFORMA program [21]. The additional clusters were located in order to obtain a denser systematic cluster design. Each cluster consisted of eight field measured sample plots with a radius of 15 m and a distance of 250 m between the plots. The cluster design as well as the plot size and field protocol were adopted from the NAFORMA program [31] apart from the fact that two plots in each cluster were removed. The removal of these plots was the result of the width of one flight line which could not cover the whole cluster.

The field measurements on the 88 circular sample plots were conducted during January–February 2014. On each plot, information such as land use, land cover, and disturbance history were recorded, and the plots were photographed. Handheld GPS receivers were used to navigate to the predefined plot centers. For the 32 previously established plots, the plot centers were found and identified according to marks placed at the first measurement [31]. On all plots, the plot center coordinates were determined by means of combined differential global positioning system (GPS) and global navigation satellite system (GLONASS) using a 40-channel dual frequency survey grade receiver as field unit. The field unit was placed in the center of each plot on a 2.9 m rod and data recording lasted for 19–55 min (mean 30 min) with a 1-s logging rate. A second receiver was used as a base station located in Liwale town. Before the positioning of the plots started, the coordinates of the base station antenna were determined with precise point positioning with GPS and GLONASS data collected continuously for 24 h, following recommendations from Kouba [32]. The distances between the plots and the base station were <76 km. Pinnacle version 1.00 post-processing software was used to compute coordinates with the base station as reference. The standard errors of the planimetric plot coordinates reported by Pinnacle ranged from 0.01 to 0.28 m with an average of 0.05 m (Additional file 1).

#### Soil carbon

Soil carbon measurements were taken from NAFORMA’s original plot numbers 4, 7 and 10 from each cluster, using the method described in NAFORMA’s biophysical field manual [31]. On the border of each soil sampling plot, four minipits were located in the four cardinal directions. At each vertical minipit wall, starting from the top, a volumetric soil sample was collected from three depths, 0–10, 10–20 and 20–30 cm. Soil samples from the respective depths were bulked into one per plot. Soils were analyzed for carbon content according to Walkley and Black [33] and bulk density [34] and then converted to ton C hectare^−1^. After removal of five outliers, 28 plots having a valid soil carbon measurement remained for modeling. The removal of measurements from five plots is unfortunate when considering the small sample, however, a boxplot analyses revealed three extreme outliers. These three samples showed SOC values over 4% and >130 ton SOC ha^−1^ in the top 30 cm of soil which is unrealistic on these predominately sandy soils. Two additional samples had Bulk Density values of <1, i.e. the density of water. These five samples were excluded as lab or sampling errors.

#### Above and below ground tree biomass

The tree measurements were acquired using concentric circular plots to define the diameter limits of trees to be included in the measurements on each part of a plot. The radii of the concentric circles were 2, 5, 10 and 15 m [21], and trees with diameter at breast height (dbh) greater than 1, 5, 10, and 20 cm, respectively, for the concentric plots of increasing size were measured. A botanist determined and recorded tree species for every tree. Every fifth tree on a plot was selected as a sample tree for height measurement using Suunto hypsometers. For trees without height measurements, tree height was predicted according to diameter-height models constructed from the sample trees. Ground reference AGB and BGB on a plot was calculated by summing individual tree biomass predictions using single-tree allometric tree-species independent models of total AGB and BGB [35] with dbh and tree height as independent variables. AGB on the plots ranged from 0 to 133.5 Mg ha^−1^ with a mean and standard deviation of 51.3 and 45.9 Mg ha^−1^, respectively. BGB on the plots ranged from 0 to 56.5 Mg ha^−1^ with a mean and standard deviation of 18.6 and 14.5 Mg ha^−1^, respectively.

Three plots with unusually high biomass were analyzed and removed after confirming that single large trees close to the plot boundary were influencing the measurements to an unproportional degree given that about half the canopy were outside the plots.

### Remotely sensed data

#### Airborne laser scanning data

The ALS data were acquired on 1 March 2014 using a Leica ALS70 laser scanner mounted on a Cessna 404 two-engine fixed-wing aircraft. Twenty-two parallel flight-lines were flown as a block with three additional flight lines perpendicular to the main direction of the block. The maximum half scan-angle was 20 degrees. The flying speed was 77 m s^−1^ and the altitude was 1200 m above ground level. The data were acquired at a pulse repetition frequency of 193.2 kHz and the resulting average pulse density on the ground was 11.9 pulses m^−2^. The data were processed and every echo was classified as “ground” or “non-ground” by the contractor (TerraTec AS, Norway) using TerraScan software and the progressive TIN densification algorithm [36]. Heights relative to the TIN surface were computed for every echo.

The software FUSION/LDV [37] was used for computation of metrics from the laser returns and a total of 66 variables were used as candidates for being included as independent variables in the regression models. The ALS metrics were computed using point elevations above ground within the 15 m radius field surveyed plots. A height threshold of 1.5 m was used for most variables to exclude sub-canopy vegetation from the tree canopies. We also calculated various ratios of all returns above 3, 5, 7.5 and 10 m. If two variables had a Pearson correlation above 0.99 or below −0.99 one of the variables was removed before modeling.

#### Landsat data

Six Landsat 8 OLI images acquired during the period 12 May and 1 September 2014 were almost cloud free over the study area. The reason for this specific time period is that the ground measurements where obtained during spring 2014 and during that year cloud free images before and after the specified dates did not exist. These six images were downloaded from USGS (http://earthexplorer.usgs.gov/). Both Standard Terrain Correction (Level 1T) OLI data and Provisional Landsat 8 Surface reflectance product images (LaSRC, version 2.2) were downloaded for further testing.

The ground control points used for Level 1T correction are derived from the GLS2000 data set (http://landsat.usgs.gov/science_GLS.php). The bands analyzed to determine which OLI scene to use were OLI bands 2–7 and NDVI which had a pixel size of 30 m × 30 m. In the final modelling three OLI band ratios (Band5/Band4, Band6/Band4, Band7/Band4) were added and therefore the statistics obtained from this initial screening of suitable image acquisitions might differ slightly from those obtained for the final models. Based on studies of the images, including analysis of correlations, scatter plots and best subset regressions with the data to be modeled as dependent variables, one of the Landsat images was selected for further modelling of SOC and another image for modeling of AGB and BGB. Image data corresponding to the field plots were extracted using bilinear interpolation.

### Regression analysis

Final models for prediction of SOC, AGB and BGB were developed using three sets of sensor data: only Landsat 8 OLI, only ALS, and the combination of Landsat 8 OLI and ALS. Of the total 79 variables used, 10 were obtained from the Landsat 8 OLI data including NDVI and three ratios that by experience is known to be of importance for biomass assessments, 59 from the ALS dataset as derived using FUSION/LDV [37], 7 from combinations of two or more of the FUSION/LDV generated ALS variables and three from the combination of ALS and Landsat 8 OLI data. When modeling forest biomass from only Landsat data, intensity data are, in particular from the mid infrared bands, often important since much of the tree-size related signal is driven by shadows [38]. Compared to spectral data, ALS data models the tree size related information better. What mainly remain to be modeled with the spectral data is thus the difference between vegetation types, which indirectly also influence the biomass. A combination of different ALS metrics, Landsat 8 OLI bands and ratios between these bands, as well as ALS metrics and spectral data, might therefore improve the regression models.

A best subset routine [39] using stepwise exhaustive search was used to select a number of models with two to six variables. Selection of the final models then depended on studies of model statistics such as Mallow’s Cp and Akaike information criterion (AIC), residual analysis and correlation analysis. We applied different log models, multiplicative models and models with square root transformed variables but in the end multiple linear regression performed as well as more complicated models.

## Results

### Selection of Landsat 8 OLI image

The usefulness of the six available Landsat 8 OLI images as well as the processing levels 1T or surface reflectance calibration (SRC) were compared using visual inspection, scatter plots, correlations and best subset regressions. Table 1 shows the results in terms of R^2^ from the best subset regressions using three explanatory variables [39].

**Table 1:** Adjusted coefficients of determination in percent [R^2^(adj), %] for best subsets regressions (italics) with three explanatory variables for six different Landsat 8 OLI images and two different processing levels

Modeled variable	Processing level	12 May 2014	13 June 2014	29 June 2014	15 July 2014	31 July 2014	1 Sept 2014
SOC	1T	8.9	19.5	20.9	21.8	30.0	30.0
SOC	SRC	11.1	19.8	19.9	20.4	*32.4*	27.9
AGB	1T	30.4	24.1	18.4	15.2	4.5	8.8
AGB	SRC	*30.5*	23.9	17.9	14.6	8.0	9.5
BGB	1T	31.8	22.4	17.7	13.7	4.0	4.6
BGB	*SRC*	*31.6*	22.6	17.5	13.9	6.8	5.1

Based on studies of the scenes, scatter plots, and the coefficients of determination presented in Table 1, we selected the Landsat 8 OLI scene from 12 May 2014, (scene id LC81660672014132LGNlarge 00), for the further modelling of AGB and BGB. This image was cloud free for 86 of the 88 plots.

We selected the Landsat 8 OLI scene from 31 July 2014 (scene id LC81660672014212LGN00) for modeling of SOC. This image was cloud free for 87 of the 88 plots. We also decided to use the surface reflectance calibrated product (SRC) only, even though the differences between the two levels of radiometric calibration did not influence the final result to a large extent.

### Final models

The final models for prediction of SOC, AGB and BGB, using only spectral information from Landsat 8 OLI SRC data, only ALS and the combination of both data sources are presented in Table 2.

**Table 2:** Results from plot level regression analysis of soil carbon (SOC), above ground tree biomass (AGB) and below ground tree biomass (BGB) using Landsat 8 OLI, ALS and the combination of these data sources

Data source	Model^a,b^	R^2^(adj)	RMSE	RMSE
		%	Mg ha^−1^	%
OLI 140731	SOC = −113.8 + 0.0637 B7 + 22.93 B5/B4	34.6	16.2	27.9
ALS	SOC = 74.97 − 0.000500 XL1 + 0.425 XL2 + 0.02500 XL3	42.4	15.2	26.2
ALS + OLI 140731	SOC = −4.2 + 36.93 B7/B4 − 0.000429 XL1 + 0.00433 XL4	56.0	13.3	22.9
OLI 140512	AGB = 35.3 − 0.0661 B6 − 18.41 B5/B4 + 55.20 B6/B4	38.1	30.6	66.2
ALS	AGB = 5.92 − 5.05 P60 + 1.248 PFR50 + 0.576 PFR75	64.4	23.3	50.3
ALS + OLI 140512	AGB = 80.8 − 0.02178 B5 − 3.38 P60 + 1.499 PFR75	66.0	22.7	49.1
OLI 140512	BGB = 39.4 − 0.02009 B5 + 7.12 B6/B4	40.1	11.6	62.2
ALS	BGB = 1.99 − 1.960 MAD − 0.943 P70 + 0.6644 PFR500	71.5	8.1	43.4
ALS + OLI 140512	BGB = 19.43 − 0.00549 B5 − 0.03186 XLS1 + 0.6417 PFR500	71.8	8.1	43.3

The R^2^_adj_ statistic is for the model and RMSE values from ‘leave one out cross validation’ (LOOCV)

XL and XLS = combination of different variables. For full variable explanation see [37]

XL1 = “return 1 count above −1.00” + “total return count above −1.00”

XL2 = “percentage first returns above 1.50”/“P80”

XL3 = “P90” * “percentage first returns above 1.50” * “return 1 count above −1.00”/“total return count above −1.00”

XL4 = “P90” * “percentage first returns above 1.50” * “return 1 count above −1.00”/“return 2 count above −1.00”

XLS1 = “percentage first returns above 1.50” * “P80”/(“NDVI + 1”); MAD = elev MAD median

^a^All regression coefficients were statistically significant at 5% level

^b^B = Landsat 8 OLI band (1, 2,…,8); P = Height percentiles of lidar vegetation echoes (0, 10,…,90); PFR = Percentage first lidar returns above heightbreak in dm (50, 75, 100)

**Fig. 5:**
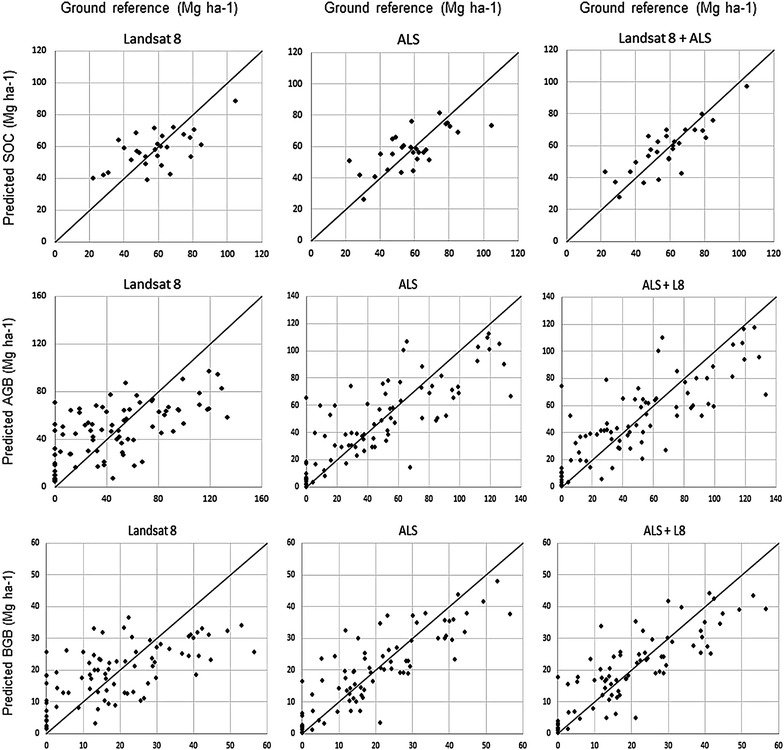
Observed versus predicted plot level SOC, AGB and BGB, using data from Landsat 8 OLI SRC, and ALS separately and in combination

Scatter plots of observed versus predicted SOC on the measured plots are shown in Fig. 5.

## Discussion

A first observation from Table 1 is that the Landsat 8 OLI data from the end of July, which is about three months into the dry season, were best for modeling SOC. On the other hand, the R^2^(adj) values for modeling AGB and BGB from OLI decreased steadily from the May 12th to the July 31st images. The Landsat 8 OLI data from May 12th were therefore best for modeling AGB and BGB. The area disturbed by recent fires also increased between July and September (Fig. 4). In the July 31st image one to four plots were disturbed by fire as seen by fire scars, and in the September 1st image the amount was between 40 and 50 plots. The number of burnt plots was extracted using visual interpretation of the satellite images and is therefore in some cases difficult to classify with certainty. The studies of MODIS and AVHRR satellite data time series have also confirmed a seasonal pattern with high values for tasseled cap greenness [40] and NDVI [41] during the rainy season. Given the increased availability of free optical satellite data, there are therefore good reasons for carefully selecting the optimal image for the given task. It is also evident from Table 1 that the standard Landsat 8 OLI level 1T data performed similarly to the ground reflectance calibrated Landsat data for the task of modeling with field plot data as independent variables.

The issue addressed in this article is to which degree the combination of Landsat 8 OLI and ALS data can improve models of AGB, BGB and SOC, compared to only using data from one of these sensors separately. It was found that modeling of AGB performed substantially better with ALS than with OLI data (Table 2). This is expected and in accordance with other studies from forest covered landscapes where ALS and optical satellite data have been compared [42, 43]. It was also found that the model for predicting AGB based on ALS was only marginally improved when adding optical satellite data as additional independent variables. This is also in accordance with earlier studies, for example Ediriweera et al. [30] who found improvements in one forest type but not in another, when Landsat TM data were added to an ALS based biomass model. The results for BGB followed the results for AGB, which is as expected since they are modeled from the same field survey of tree stems.

A unique finding is that ALS data that describe the forest and woodland canopy could also be used for modeling of soil carbon. In this study, ALS data were superior to Landsat 8 OLI for modeling SOC. The combination of ALS and Landsat 8 OLI data further improved the models obtained in comparison with either of these sensors separately. A reason for this is that ALS is superior to two-dimensional optical satellite data for the purpose of modeling tree biomass, and SOC generally is positively correlated with tree cover [11]. It is also logical that the spectral data provide additional information when combined with ALS, since the spectral data will contribute with both information about soil colour and field layer vegetation that is not captured by the ALS point cloud. There are only a few studies where SOC has been modeled based on tree cover data from ALS. Kristensen et al. [44] tested this at a site in a boreal forest in southern Norway and found a weak correlation between tree canopy density and height obtained from ALS and the organic layer C stock. They found a stronger correlation with organic layer C stock and topographical wetness index obtained from the ALS based elevation model. The use of terrain variables for modeling SOC was however not tested in the present study.

Vågen and Winowiecki [17], Vagen et al. [45] and Winowiecki et al. [11] obtained even higher R^2^ values when modeling SOC with optical satellite data than obtained in the present study. The reasons contributing to this might be that they used larger plots with more soil samples per plot, and that they had many more field plots which allowed development of more complex models as well as enabling the inclusion of a greater span of data, which tends to improve R^2^. The importance of a large plot size was also noted by Mauya et al. [29] who showed that when using ALS data for predicting AGB in a tropical rain forest of Tanzania R2 increased from around 0.4 for 700 m^2^ plots to around 0.75 for 2000 m^2^ plots. The plot radius 15 m used in this study was mainly because we used the same field instructions as in the NAFORMA inventory. The fact that three plots had to be omitted because of single large trees near the plot borders indicate however that much larger plots might be needed in the woodlands of Africa than in the boreal where plot radius of about 10 m most often are used.

When modeling AGB with OLI, we obtained plot level R^2^(adj) of 38% and RMSE 63% using three explanatory variables. The results for modeling of BGB were similar. As a comparison, Gizachew et al. [28] obtained a plot level RMSE of 49% for AGB + BGB by using only NDVI from an OLI image acquired over Liwale on 31 July 2014. Their study area was 15,700 km^2^ in size and covered a substantial part of the Liwale district and their model was trained with 500 plots from the original NAFORMA inventory, which is stratified for tree biomass [21]. Two of the three explanatory variables we used for modeling AGB with OLI contained short wave infrared (SWIR) bands. These bands are missing on some remote sensing sensors, and their importance for the modeling of forest biomass was already noted when Landsat TM was new [38]. One reason for their importance for forest biomass assessment is probably that the shadows from the trees are more evident in these bands [38]. It should however be observed that the high solar angles in the tropics reduces the effect of tree shadows, and care should therefore be taken when transferring research results about optical forest remote sensing from other latitudes.

Næsset et al. [22] used the same field plots as in the current study, as part of a study regarding remote sensing data as an aid in large area sampling. They obtained the same RMSE, 63%, for modeling of AGB with RapidEye data as we obtained with OLI in this study, while their results with InSAR, Global tree cover maps from Landsat, and PALSAR products were slightly less good. Their model of AGB using ALS has about the same R^2^, but the RMSE is slightly lower in the present study, probably since outliers were removed in this study. Our model for AGB with ALS is also very similar to the accuracy and R^2^ obtained by Mauya et al. [29] who modeled AGB with ALS over the larger 15,700 km^2^ area in Liwale, using plots from the NAFORMA inventory.

There are several sources of error that should be noted. The number of available field plots was limited, especially since only 28 plots were used for modeling SOC. Of the 88 plots with tree biomass measurements, three were not used because large trees near the plot boundary considerably disturbed the relationship with the remote sensing data. Two additional plots were cloud covered in the satellite image used for the biomass modeling. We tried to avoid overfitting by using simple regression functions with few explanatory variables.

Another source of uncertainty that may have affected the model fit is the sub-sampling of trees within a plot as described by the field protocol [31]. The smaller trees were only recorded in the center of each plot, and for a radius of >10 m (the outer 393 m^2^ of each plot) only trees with dbh >20 cm were recorded. The amount of biomass for the smaller trees was estimated from the recordings in the inner parts of the plots. When inspecting the plots visually using the ALS point clouds, we noticed some plots in which smaller trees were present in the outer part of some plots for which ALS echoes were included in the AGB prediction for the plot, while no or only a few trees had been recorded in field. To take full advantage of ALS data to improve forest parameter estimates, field protocols should reflect the utility of measuring the same trees on the ground as observed by the remote sensor.

The overall conclusions from this study are that SOC, AGB and BGB can be modeled in Miombo woodlands and forests with Landsat 8 OLI and similar satellite data such as from Sentinel 2 or SPOT, but that even better results are obtained when using ALS data. However, the best results were obtained by combining Landsat 8 OLI data and ALS, in particular when modeling SOC.

## Additional file

**Additional file 1.** PlotData.

## Abbreviations

AGB: above ground tree biomass
ALS: airborne laser scanning
BGB: below ground tree biomass
GLONASS: global navigation satellite system
GPS: global positioning system
MRV: measuring, reporting and verification
NAFORMA: National Forest Resources Monitoring and Assessment of Tanzania
NDVI: Normalized Difference Vegetation Index
OLI: Operational Land Imager
REDD+: reduce emissions from deforestation and forest degradation
SOC: soil organic carbon
SRC: surface reflectance calibrated
UNFCCC: United Nations Framework Convention on Climate Change
